# A Review on Real-Time 3D Ultrasound Imaging Technology

**DOI:** 10.1155/2017/6027029

**Published:** 2017-03-26

**Authors:** Qinghua Huang, Zhaozheng Zeng

**Affiliations:** ^1^School of Electronic and Information Engineering, South China University of Technology, Guangzhou, China; ^2^College of Information Engineering, Shenzhen University, Shenzhen 518060, China

## Abstract

Real-time three-dimensional (3D) ultrasound (US) has attracted much more attention in medical researches because it provides interactive feedback to help clinicians acquire high-quality images as well as timely spatial information of the scanned area and hence is necessary in intraoperative ultrasound examinations. Plenty of publications have been declared to complete the real-time or near real-time visualization of 3D ultrasound using volumetric probes or the routinely used two-dimensional (2D) probes. So far, a review on how to design an interactive system with appropriate processing algorithms remains missing, resulting in the lack of systematic understanding of the relevant technology. In this article, previous and the latest work on designing a real-time or near real-time 3D ultrasound imaging system are reviewed. Specifically, the data acquisition techniques, reconstruction algorithms, volume rendering methods, and clinical applications are presented. Moreover, the advantages and disadvantages of state-of-the-art approaches are discussed in detail.

## 1. Introduction

Many imaging technologies have been applied to enhance clinicians' ability for diagnosis of the disease, for example, the X-ray, magnetic resonance (MR), computed tomography (CT), and ultrasound (US). Each imaging modality has its strengths and limitations in different applications [1]. Among these diagnosis-aid technologies, US gains more and more attention in recent years. Aside from low cost and no radiation, the interactive nature of US which is mostly needed in surgery facilitates its widespread use in clinical practices.

Conventional 2D US has been widely used because it can dynamically display 2D images of the region of interest (ROI) in real-time [2, 3]. However, due to the lack of the anatomy and orientation information, clinicians have to imagine the volume with the planar 2D images mentally when they need the view of 3D anatomic structures. The limitation of 2D US imaging makes the diagnostic accuracy much uncertain as it heavily depends on the experience and knowledge of clinicians. In order to address the foresaid problem, 3D US was proposed to help the diagnosticians acquire a full understanding of the spatial anatomic relationship. Physicians can view arbitrary plane of the reconstructed 3D volume as well as panoramic view of the ROI which helps surgeons to ascertain whether a surgical instrument is placed correctly within the ROI or just locates peripherally during the surgery [4]. It is undeniable that 3D US enables clinicians to diagnose fast and accurately as it reduces the time spent on evaluating images and interacts with diagnosticians friendly to obtain a handle of the shape and location of the lesion.

Generally, 3D US imaging can be conducted with three main stages: that is, acquisition, reconstruction, and visualization. The acquisition refers to collecting the B-scans with relative position using conventional 2D probes or directly obtaining 3D images using dedicated 3D probes. The reconstruction aims to insert the collected 2D images into a predefined regular volume grid. The visualization is to render the built voxel array in a certain manner like any-plane slicing, surface rendering, or volume rendering. Traditional 3D US is temporally separated into the B-scan frame collection, volume reconstruction, and visualization stages individually, making it time-consuming and inefficient to obtain an accurate 3D image. Clinician has to wait for the data collection and volume reconstruction which often take several minutes or even longer time before visualizing any part of the volume, rather than visualizing 3D anatomy simultaneously during the scanning of the ROI. Hence the clinician cannot select an optimal way to conduct the scanning process for subsequent diagnosis. Moreover, the separation has limited the applications in surgery where physicians require immediate feedback on intraoperative changes in the ROI [5]. It is no doubt that real-time 3D US will facilitate physicians' ability in diagnosis even better and help them work more efficiently during the surgery.

Many investigators have made their efforts to develop the real-time or near real-time US systems in recent decade. Several attempts with the dedicated 3D probe or traditional 2D probe to reconstruct and render a volume during data acquisition are now available. To provide systematic understanding of the relevant technology in real-time US, we review the state-of-the-art approaches for designing real-time or near real-time 3D US imaging system. Data acquisition techniques, reconstruction algorithms, rendering methods, and clinical applications are discussed in the following sections, including the advantage and disadvantages of each approach.

## 2. Data Acquisition

Obtaining 3D real-time US image without distortions is crucial for the subsequent clinical diagnosis. In any approach of data acquisition, the objectives are twofold: first to acquire relative locations and orientations of the tomographic images accurately, which ensures the 3D reconstruction without errors, and second to capture the ROI expeditiously, which is aimed at avoiding the artifacts caused by cardiac, respiratory, and involuntary motion, as well as enabling the 3D visualization of dynamic structures in real-time. Four representative real-time 3D US data acquisition techniques have been proposed, that is, 2D array transducers, mechanical 3D probes, mechanical localizers, and freehand scanners.

### 2.1. 2D Array Transducers

In conventional 1D array transducer, a subset of transducer elements or subaperture is sequentially selected to send an acoustic beam perpendicularly to the transducer surface, and one line is drawn at the same time. Through multiplexing or simply turning elements on and off, the entire aperture can be selected which forms a rectangular scan [6]. Analogously, 2D array transducers derive an acoustic beam steering in both azimuth and elevation dimensions, which enables obtaining a volumetric scan [7].

**Figure 1:**
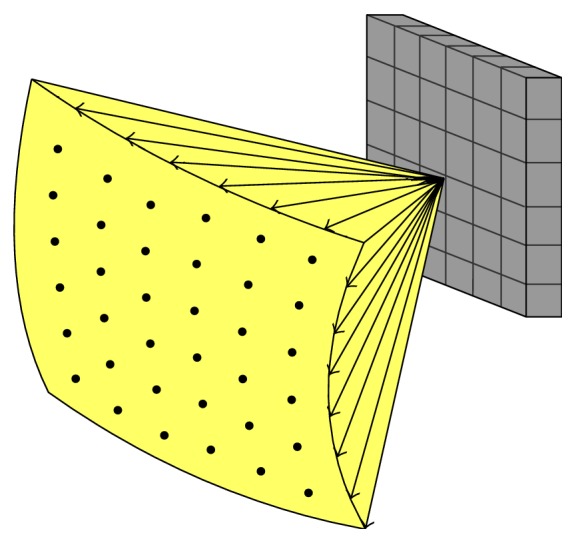
Principle of volumetric imaging with a 2D array transducer.

2D array transducers acquire 3D information by electronic scanning. As illustrated in Figure 1, the elements of 2D array transducer generate a diverging beam in a pyramidal shape and the received echoes are processed to integrate 3D US images in real-time. Since the beams can be steered and focused on the ROI by adjusting the phased array delays [8], the transducers can remain stationary while being used to scan.

A variety of 2D array patterns are proposed to fabricate 2D array transducers, such as sparse periodic array, Mills cross array [9], random array, and Vernier array. As 1D linear transducers, 2D array transducers can be sorted of concave surface and flat surface. Concave transducers have an advantage of concentrating a higher energy to the focal areas. Flat transducers have a wider steerable area of acoustic field [10]. The elements of 2D array transducers can be arranged as either a rectangle or an annular array [11].

**Figure 2:**
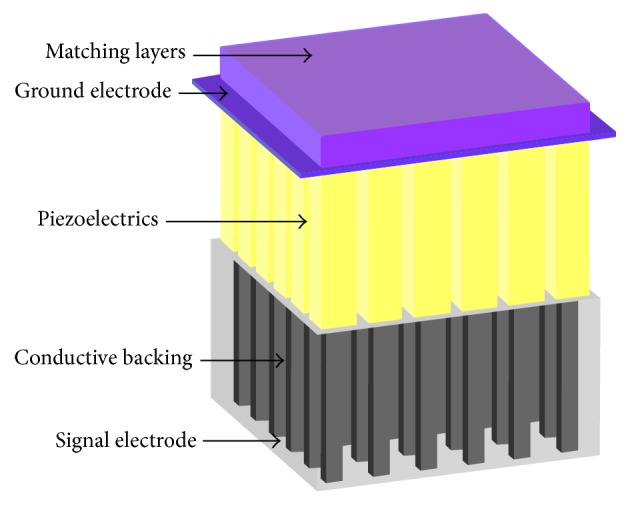
The consisting material of a single-unit type matrix transducer.

The substrates of 2D array transducers can be fabricated with various piezoelectric materials (Figure 2), such as lead zirconate titanate (PZT), lead magnesium niobate–lead titanate (PMN–PT), and piezocomposites [12]. Aside from piezoelectric transducers, capacitive micromachined US transducers (CMUTs) have also shown a potential performance as their counterparts [13].

Since the concept of 2D array transducers was proposed by Duke University in 1990s, various researchers and commercial companies are concentrated on the development of 2D array transducers. The real-time performance and fabrication parameters of several typical 2D array transducers are listed in Table 1.

Although 2D array transducers are capable of realizing the 3D visualization of dynamic structures in real-time directly and ideally, the electrical impedance of each element in 2D array transducers is much greater than that in 1D array transducers, which makes impedance matching of 2D array elements challenging [14]. Furthermore, to avoid the cross-talk between elements, a half-wavelength distance is needed for the neighbor elements, which results in a large number of elements and extremely small size of each element. To reduce the difficulties in fabrication of 2D array transducer, the size of the array cannot be large, which leads to a small field of view in imaging. Several problems should be resolved before 2D array transducer becoming widespread in clinical examinations.

### 2.2. Mechanical 3D Probes

**Figure 3:**
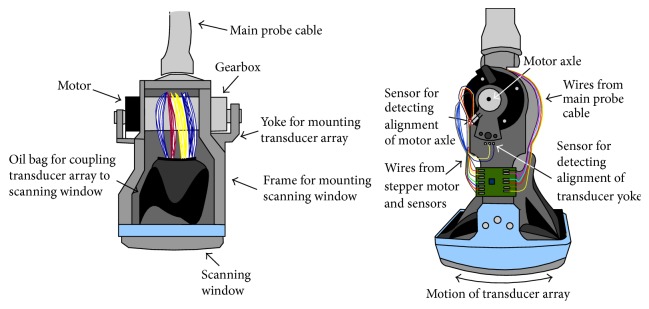
Schematic structure of a mechanical 3D probe.

**Figure 4:**
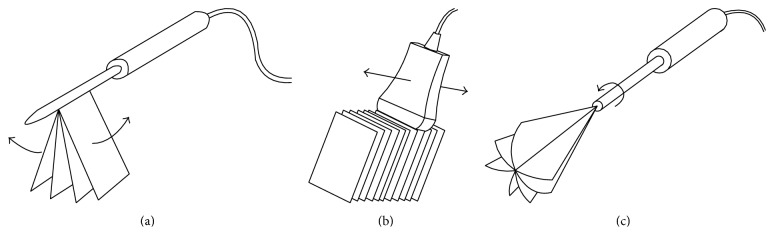
Schematic structure of three types of mechanical scanning: (a) tilting scanning; (b) linear scanning; (c) rotational scanning.

Other 3D probes are developed for real-time 3D US imaging by assembling a linear array transducer inside a handheld instrument (Figure 3). In a mechanical 3D probe, a regular linear array transducer is motored to rotate, tilt, or translate within the probe under the computer control [15]. Multiple 2D images are acquired over the examined area when the motor is activated [16]. The axis of rotation, tilt, or translation can be used as reference frame for 3D images reconstruction. Three types of mechanical scanning are illustrated in Figure 4, that is, linear scanning, tilting scanning, and rotational scanning.

#### 2.2.1. Linear Scanning

In this approach, the transducer is driven by a mechanism to translate across the ROI. The scanning route of the transducer is parallel to the surface of the skin and perpendicular to the image plane. The acquired images are parallel and equidistantly spaced and their spacing interval can be adjusted by changing the image frame rate. The resolution of 3D images produced by this approach is not isotropic. The resolutions in the directions parallel to acquired 2D image planes are the same as the original 2D images, and the resolution in the direction of scanning route depends on the elevational resolution of mechanical 3D probe.

#### 2.2.2. Tilting Scanning

In the tilting scanning, the transducer is motored to tilt about an axis at the transduce surface. A fan of planes is acquired and the angular separation between images is adjustable, which depends on the rotational speed of motors and the image frame rate. When acquiring images, the probe should be fixed on the skin of patients. The resolution of produced 3D images is not isotropic which degrades as the distance from the tilt axis increases. The time of obtaining 3D volume depends on image update rate and the quantity of the required images.

#### 2.2.3. Rotational Scanning

In rotational scanning method, the transducer is driven to rotate with central axis of the probe. The axis should remain fixed when the ROI is being scanned. The rotational scanning probe is sensitive to the motion of transducer such that resulting 3D images will contain artifacts if any motion occurs during the scan. The resolution of the obtained 3D images is also not isotropic. The resolution will degrade as the distance from the axis increases. If a convex transducer is assembled in the probe, the corresponding resulting 3D images will be in a conical shape; otherwise, a cylinder will be obtained when a flat transducer is employed.

#### 2.2.4. Summary

For various applications in clinical practice, a variety of mechanical 3D probes are developed in recent decades. For instance, Downey and Fenster [17] proposed a real-time 3D US imaging system which consists of rotating and linear mechanical 3D probes for different applications. The sequence of images can be acquired at 12.5 frames/s and reconstructed immediately. The system has been applied in breast, prostate, and vascular examination, and the acquisition resolution can be set as 0.3–1.0 mm depending on the ROI.

Mechanical 3D probes are made compactly and they are convenient to operate, though they are comparatively larger than conventional linear probes. The needed imaging and reconstruction time is short which enables viewing high-quality 3D images in real-time. However, clinicians are required to hold the mechanical 3D probes statically while acquiring images, which will lead to latent errors for data acquisition. Furthermore, a particular mechanical motor is needed for integrating with transducer, which is lack of universality.

### 2.3. Mechanical Localizers

Similar to mechanical 3D probes, mechanical localizers are driven by motorized mechanisms. In a 3D mechanical probe, the scanning mechanism is integrated inside a handheld instrument together with a special 1D linear transducer. Nevertheless, a mechanical localizer consists of an external fixture which holds a conventional 1D transducer to acquire a series of sequential 2D images [18, 19].

Generally, the scanning route is predefined such that the relative positions and orientations of acquired 2D images can be precisely recorded in computers. With this location information, 3D US images can be reconstruction in real-time. The angular and spacing interval between each frame can be adjusted to obtain optimal resolution and minimize the scanning time. Similar to mechanical 3D probes, the patterns of mechanical localizers scanning can be grouped into 3 types: that is, linear, tilt, and rotation.

Several mechanical localizers systems have been proposed for real-time 3D US imaging, such as Life Imaging System L3Di 3D US acquisition system, which can drive probes in linear scanning for carotid arteries diagnosis [20]. The mechanical localizers have capacity of holding any conventional transducers such that they can undertake the developed US imaging probes without any update to themselves [21]. However, the mechanical localizers are always enormous and heavy, making them inconvenient in applications.

### 2.4. Freehand Scanners

**Figure 5:**
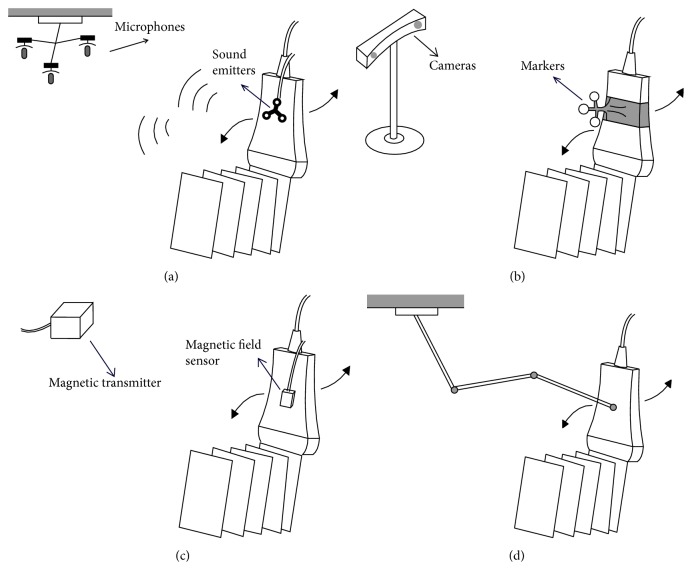
Schematic structure of three types of position sensor: (a) acoustic sensor; (b) optimal positioner; (c) magnetic field sensor; (d) articulated arm positioner.

Obviating the need for cumbersome mechanism, freehand scanners are flexible and convenient to operate. Using a freehand scanner, clinicians can scan the ROI in arbitrary directions and positions, enabling clinicians to choose optimal views and accommodate complexity of anatomy surface. Positions and orientations of 2D B-scans are needed for reconstructing 3D images. Four approaches with different positional sensors were proposed for tracking the US probe: that is, acoustic positioner, optical positioner, articulated arm positioner, and magnetic field sensor (Figure 5). In addition, image-based approaches without positional sensors were also developed, for example, speckle decorrelation.

#### 2.4.1. Acoustic Positioner

In this approach, three sound emitting devices are mounted fixedly on the transducer, and an array of microphones is placed over the patient. The microphones receive acoustic wave continuously from sound emitters during the scanning. Positions and orientations can be calculated for reconstructing 3D images with knowledge of the speed of sound in air, the measured time-of-flight from each sound emitter to microphones, and the positions of microphones [22]. To guarantee a good signal-to-noise ratio (SNR), microphones should be placed closely to the patients and the space between emitters and microphones should be free of obstacles.

#### 2.4.2. Optical Positioner

A freehand transducer with optical positioner system consists of passive or active targets fixed on the transducer and at least two cameras used to track targets. By observing targets from 2D images, the position and orientation can be calculated with knowledge of relative positions of targets [23]. Optical positioners can be divided into passive stereovision system and active marker system. Passive stereovision systems make use of three or more matt objects as targets and active marker system utilizes several infrared diodes as markers, whose frequency is already known. A freehand transducer with optical positioner is stable and has high accuracy.

#### 2.4.3. Articulated Arm Positioner

In this approach, a transducer is mounted on an articulated arm with multiple movable joints. Unlike mechanical localizer, clinicians can manipulate the transducer with an articulated arm positioner in arbitrary orientations to obtain optimal views. Potentiometers located on the joints can monitor the moving angulation and position of articulated arms continuously, which are effective for calculating the spacing information of transducer for 3D reconstruction. To improve the precision, the individual arms should be as short as possible, which will lead to a small range of view.

#### 2.4.4. Magnetic Field Sensor

A transducer with magnetic field sensor consists of a time-varying magnetic transmitter placed near the patient and a receiver containing three orthogonal coins attached on the transducer. The receiver measures the strength of magnetic field in three orthogonal directions; then the position and orientation of the transducer can be calculated, which is needed for 3D reconstruction. Magnetic field sensors are relatively small and more flexible without a need for unobstructed sight. However, electromagnetic interference and existence of metallic objects may compromise the tracking accuracy and cause distortion. Furthermore, to avoid tracking errors, the magnetic field sampling rate should be increased.

#### 2.4.5. Image-Based Sensing

Image-based sensing approach extracts the relative positions by analyzing the image feature, for example, speckles, instead of depending on position sensors [24]. According to the phenomenon of speckle decorrelation, the speckle pattern should be the same if two images are acquired at the same position, which results in nondecorrelation. However, the decorrelation is proportional to the distance between two images. To obtain the relative translation and rotation between two images, the acquired images are divided into small subregions. Calculated decorrelation values can be used to analyze the relative position and orientation of adjacent 2D images. Using this scanning protocol, operators are supposed to move the transducer at a constant velocity in linear or rotational manners to guarantee appropriate intervals. However, this approach is lacking accuracy.

#### 2.4.6. Summary

Welch et al. [23] proposed a real-time freehand 3D US system for image-guided surgery which utilized a 5 MHz linear transducer and an optical positioner to track the location and orientation. With the frame rate at 15 frames/s, the system was able to dynamically reconstruct, update, and render 3D volumes. Prager et al. [25] implemented volume measurement and visualization in real-time using a freehand US system with a magnetic field position sensor. With the help of optimized sequential algorithms, the 3D US volume could be resliced at 10 Hz. Dai et al. [26] developed a real-time freehand 3D US system which enabled us to semiautomatically determine the ROI using a 3.5 MHz concave probe and an electromagnetic position sensor. The system was capable of fast predetermining the reconstruction volume and assigning the optimal viewing direction, which achieved an accurate and fast reconstruction in real-time.

Without the predefined route, the freehand scanners should be moved over the skin surfaces in an appropriate speed to avoid significant gaps. Considering the variance of the environment and sensor positions, freehand scanner systems with position sensors should be calibrated every time before being used [27]. Usually, spatial calibration and time calibration are needed for calculating a spatial correction and time delay.

## 3. Reconstruction Algorithms

Aside from quality and rate of data acquisition, the speed and accuracy of volume reconstruction are significant for realizing real-time 3D US imaging. Various reconstruction algorithms were proposed for visualizing ROI simultaneously while scanning, most of which were based on the conventional 3D reconstruction algorithms and utilized parallel computing technique. Hence, reconstruction algorithms which have already completed or are potential for real-time visualization are introduced in this section. The real-time reconstruction algorithms of 3D voxel representation can be classified into 3 types based on implementation: that is, Voxel-Based Methods (VBMs), Pixel-Based Methods (PBMs), and Function-Based Methods (FBMs). The voxel value in the grid using methods mentioned above depends on the source pixels from the acquired 2D B-scan images. In the following illustrations, the 3D voxel grids are showed as 2D grids marking the centers of the voxels and the 2D input images are illustrated as lines where the points illustrate the centers of the pixels.

### 3.1. Voxel-Based Methods

In VBMs, every voxel in the predefined structured volume is traversed and assigned a value depending on one pixel or more from the acquired B-scans.

**Figure 6:**
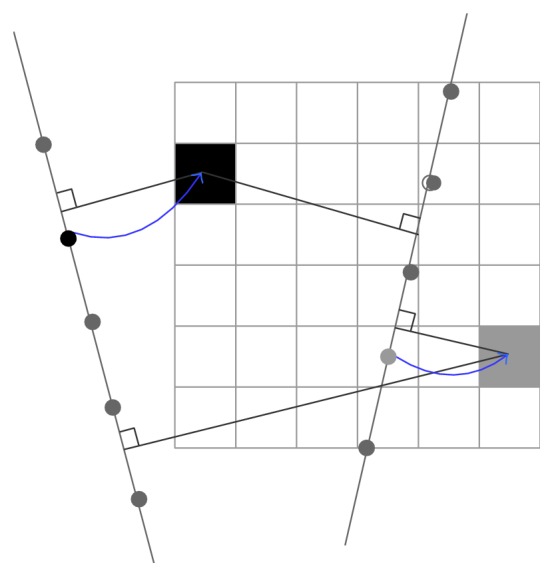
VNN. A normal from the voxel to two nearest frames is calculated and the nearest pixel is selected to be mapped into the voxel.

**Figure 7:**
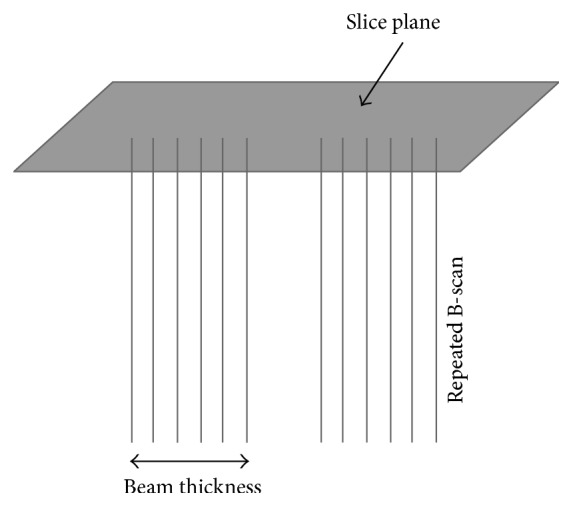
Consider the thickness of the US beam to improve inserted quality.

*One Voxel with Support of One Pixel.* The most popular one-pixel contribution is the Voxel Nearest Neighbor (VNN) [28] with a simple concept that each voxel is assigned the value of the nearest pixel from source 2D image (Figure 6). By taking into account the fact that the nearest pixel to the voxel lies on its normal to the nearest B-scan, the reconstruction can be speeded up rapidly [29]. Additionally, a volumeless method was proposed by Prager et al. [25] to produce arbitrary 2D slice through the origin data set. It traverses the pixels of the selected volume slice and maps the relative nearest pixels on the acquired frames to the slice by considering the fact that the US beam has a thickness to improve the quality of the slice (Figure 7). The system can generate planar and nonplanar slices quickly for it does not need to construct a volume grid.

**Figure 8:**
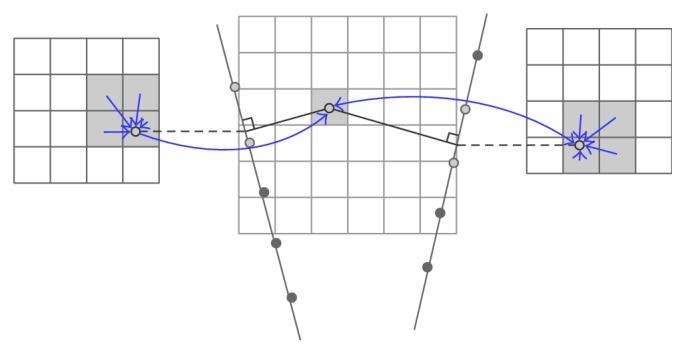
VBM with interpolation from the two nearest surrounding images where a normal to each image is calculated.

**Figure 9:**
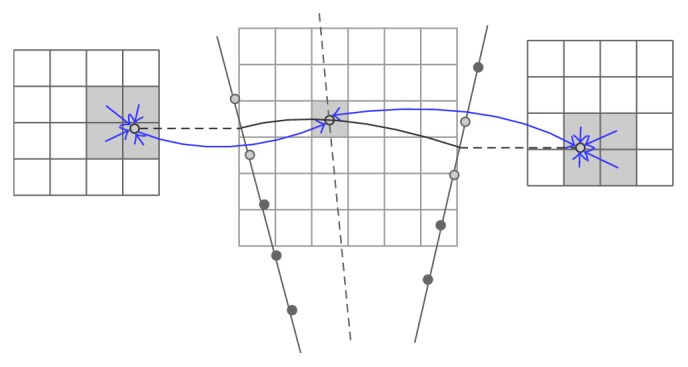
The probe trajectory used to find the two intersecting points on the two surrounding images is estimated.

*Voxel-Based Methods with Interpolation (VBMI).* The voxel value relies on the interpolation between several corresponding pixels of the captured frames. The interpolation methods that are popularly used refer to the distance weighted (DW) and its modified versions. The key concept of the DW is that the voxel value is assigned the weighted average of pixels in local neighborhood and the weight is often the inverse of the distance from the pixel to the voxel. Trobaugh et al. [30] proposed a method to traverse each voxel and search the closest two 2D B-scans on each side of the voxel. Then a normal to each surrounding B-scan was determined, passing through the voxel, to obtain the contact points on the two scans. The intensity value of the point was assigned by the bilinear interpolation of the four enclosing pixels on the scan. Afterwards, the target voxel had the value as a weighted average of the two contact points where the weight relied on the distance from the voxel to the scan plane (Figure 8). Another clever algorithm based on Trobaugh's was introduced by Coupé et al. [31] which estimated the probe trajectory between the two nearest B-scans to find intersecting points on the two planes corresponding to the current traversed voxel which was then assigned the weight sum of the two points. As what Trobaugh did, the value of the intersecting points came from the bilinear interpolation of the four closest pixels (Figure 9).

### 3.2. Pixel-Based Methods

PBMs are popular in most of the 3D US systems. They traverse the pixels of the acquired B-scans and attribute the pixel value to one or more voxels. There are some factors that result in gaps in the voxel array; for example, a sparse scanning or the voxel size is set small compared to the distance between the B-scans. Thus, a subsequent step is necessarily needed to fill the gaps. The basic algorithm mainly consists of two stages: a distribution stage (DS) and a gap-filling stage (GFS). In the DS, a current traversed pixel distributes pixel value to the nearest voxel or voxels in a definitive region with a weight value. After the DS step, gaps may occur in practice. Thus, the second stage, that is, GFS, has to fill the remaining gaps to get a desired result. We summarize algorithms for the two stages in the following.

#### 3.2.1. DS

Pixel nearest neighbor interpolation (PNN) may be the earliest and simplest reconstruction algorithm as it just fills the pixel value to the nearest voxel in the volume. If more than one pixel runs through the voxel, then the voxel value can be the average (Nelson and Pretorius [32], Gobbi and Peters [33]), maximum value (Nelson and Pretorius [32]), the most recent value (Ohbuchi et al. [34]), or the first value (Trobaugh et al. [30]) of the pixels.

Other investigators proposed some comparatively complex but improved interpolation algorithms for more accurate imaging [35–37]. These methods introduce a local neighborhood called kernel around the pixel to distribute the pixel value to the contained voxels. Every voxel accumulates the pixel values as well as the weight values which are then used to calculate the final voxel value. Thus, we can call these methods kernel-based algorithms, and some parameters, such as the weight function and the size and shape of the neighborhood, should be set prior to reconstruction.

The most commonly referred example of kernel-based algorithms is introduced by Barry et al. [38], who used a spherical kernel of radius *R* around the pixel with the weight, that is, the inverse distance. Any voxel lying in the neighborhood stores accumulated intensity contribution and relative weight from the central pixel. After traversing all the pixels, the final voxel value is computed by dividing its accumulated pixel intensity value by its accumulated weight value. It should be noted that the radius *R* influences mostly the DS result. Small *R* results in quantity of gaps, and large *R* leads to a highly smoothed volume.

**Figure 10:**
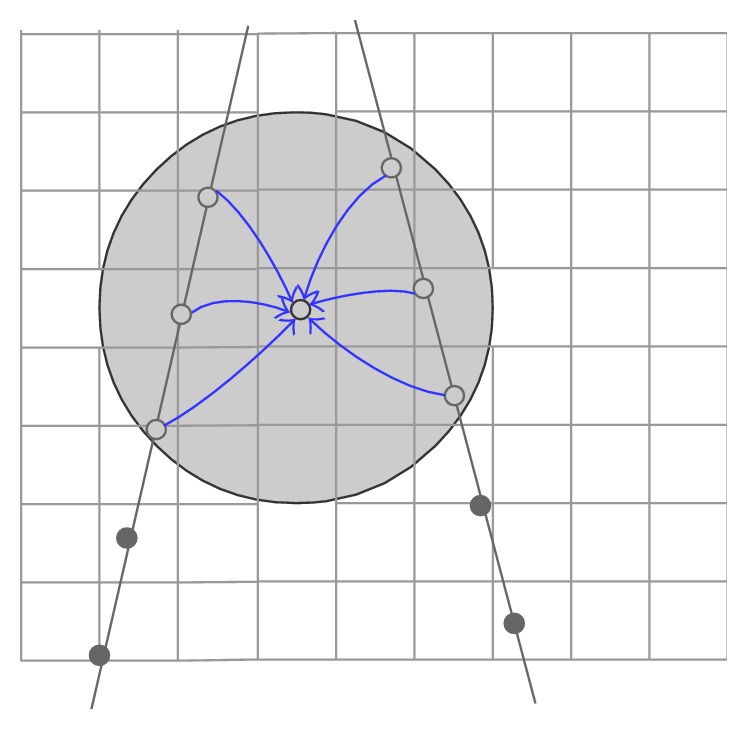
Squared distance weighted interpolation. Pixels that fall within the spherical region make value contribution to the central voxel.

Huang et al. [39] further improved the approach by introducing a positive parameter for the weight and the method is called the squared distance weighted (SDW) interpolation (Figure 10). The algorithm can be described as follows:

(1) IV→C=∑k=0nWkIV→Pk∑k=0nWk,Wk=1dk+α2,

where $I\left({\vec{V}}_{C}\right)$ is the intensity value of the target central voxel, *n* refers to the number of pixels that fall within the predefined region, $I\left({\vec{V}}_{P}^{k}\right)$ denotes the intensity of the *k*th pixel that transformed to locate on the volume *C* coordinate, while *W*_*k*_ is the relative weight for the *k*th pixel depends on the distance *d*_*k*_ from the *k*th pixel to the target voxel at *C* coordinate, and the positive parameter *α* is used to adjust the effect of the interpolation. The method can reduce the blurring effect in the 3D image since it offers the nonlinear assignment for the weights [39]. In addition, Huang and Zheng [40] proposed an adaptive strategy, namely, adaptive squared distance weighted (ASDW) method, to automatically adjust *α* by utilizing the local statistics of pixels in the spherical region around the target voxel with the goal to preserve tissue edges and reduce speckles in the 3D US image. Another adaptive method based on Gaussian convolution kernel, that is, adaptive Gaussian distance weighted (AGDW), is designed by Huang et al. [41], which performs well in speckle reduction and edges preservation as well. The simulation results show the adaptive process offers a good trade-off between the edges preservation and speckle suppression [42]. To reduce the interpolation errors, four median-filter-based methods are also proposed by Huang and Zheng [43] for calculating the voxel intensities.

Additionally, the kernel also can be cubic; for example, Gobbi and Peters [33] introduced the pixel trilinear interpolation (PTL) that made each pixel smeared into a 2 × 2 × 2 kernel and then compounded or alpha-blended into the resulting volume at an appropriate location. The compounding approach used an accumulated buffer to accumulate the weights, indicating how much the voxel was impacted by the intersected pixels while the alpha-blending method put higher weight on the newly inserted pixel than the previous ones without using the accumulated buffer for efficient computation. The compounding method can be explained as the following formulas:

(2) Ikvoxel=bkIpixel+akIkvoxelbk+ak,ak=ak+bk,

where *I*_*k*_voxel__ denotes the *k*th voxel in the volume, *I*_pixel_ means the pixel value on the B-scan, and the splat kernel coefficient *b*_*k*_ indicates how much the pixel impacts the voxel and the *a*_*k*_ accumulated weight for the corresponding voxel. The compounding method provides average of the new splat with the previous splat to reduce the noise [33].

The alpha-blending method uses the same equation that is used for image compositing via alpha blending as follows:

(3) $$\begin{array}{c} I_{k_{voxel}}=b_{k}I_{pixel}+\left(\;1-b_{k}\;\right)I_{k_{voxel}}. \end{array}$$

The additional initial condition requires that the initial voxel value *I*_*k*_voxel__ = 0. The voxel gets value unless it is hit by the splat and the first time would be *I*_*k*_voxel__ = *I*_pixel_. In this method a new pixel obscures the contribution of the previous pixels and the scheme achieves faster reconstruction compared to the compounding one.

**Figure 11:**
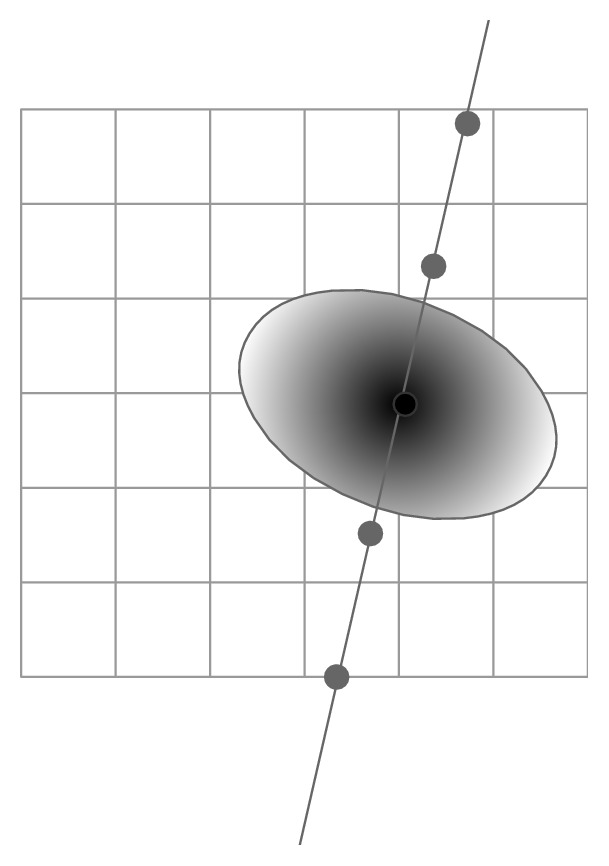
PBMs DS with a 3D ellipsoid Gaussian kernel around the pixel and the extent and weighting is determined by an ellipsoid Gaussian kernel.

Some other kernel shapes have also been availably proposed to reduce the reconstruction error and improve 3D images. By taking into account the asymmetric shape of the point spread function of the US beam, Ohbuchi et al. [34] applied an ellipsoid Gaussian convolution kernel to the neighboring pixels of each voxel (Figure 11). By assigning more weight to the most recent pixel, the change during sweeping can be taken into consideration.

#### 3.2.2. GFS

**Figure 12:**
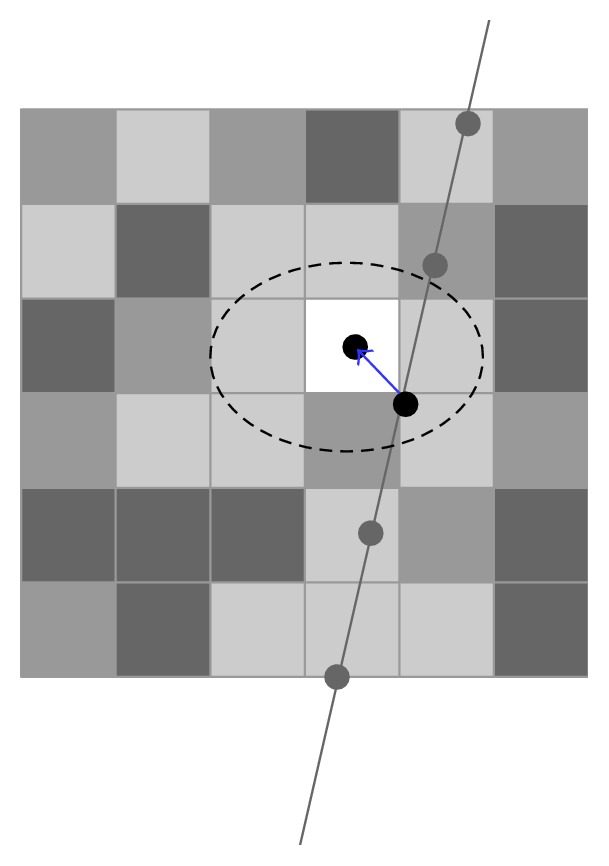
PBM GFS. Gap-filling with an ellipsoid kernel around a voxel, and the PSF of the US system is used to determine the kernel shape and weighting.

After the DS, some gaps occur in the volume array if the size of the voxel or the local neighborhood is small compared to the distance between the acquired B-scans. Therefore a necessary processing, that is, GFS, is performed to fill the empty voxels to make the volume integrated and continuous. A variety of filled strategies have been proposed; for example, Hottier and Billon [44] traversed the volume voxels and applied bilinear interpolation between two closest nonempty voxels in the transverse direction to the B-scans to the empty voxel. Other investigators applied a kernel to the filled or empty voxel, and the kernel shape can be sphere or ellipsoid and so on. Some simple interpolation strategies include replacing the hole with a nearest nonempty voxel, an average (Nelson and Pretorius [32]) or a median (Estépar et al. [45]) of the filled voxels in a local neighborhood. Other hole-filling methods already existing with more reasonable filling are of great computational cost. Huang et al. [39] enlarged the spherical region of the empty voxels to include more voxels for calculating the weighted average value using SDW for the empty voxel and the voxel value is left zero if the region size exceeds a preset threshold [39]. Estépar et al. [45] applied a normalized convolution with an ellipsoid kernel whose shape and weighting depended on point spread function of the US system to the filled voxels instead of the empty ones to complete the hole-filling (Figure 12). In the kernel methods, it is an important issue to determine the kernel size. The size can be set arbitrarily great to fill all the empty voxels but brings a highly smooth volume. Otherwise, if the size is set small, there still exist gaps in the reconstructed volume after the hole-filling. However, it is reasonable to leave the holes unprocessed, indicating that the scanning sweep has missed those locations.

It should be noted that the GFS is not necessarily needed in some situations like scanning densely or taking into account the thickness of the US beam theoretically. However, it is safe to perform the additional gap-filling to acquire an integrated result since the physicians scan arbitrarily in practice without the guarantee of dense scans.

### 3.3. Function-Based Methods (FBM)

**Figure 13:**
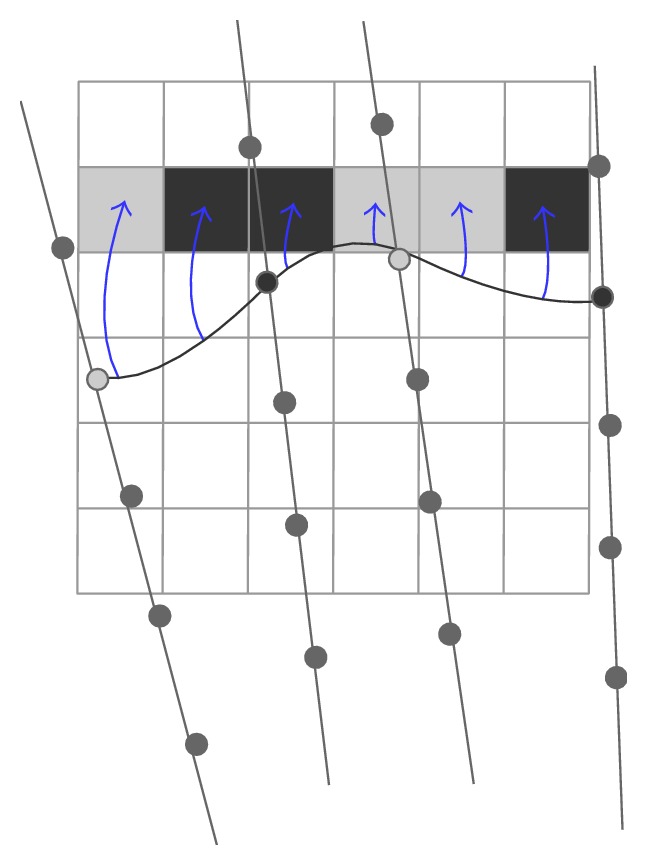
Functional interpolation. The function through the input points is estimated and evaluated at regular intervals to obtain the final voxel values.

The FBMs attempt to introduce functional interpolation for 3D US reconstruction. It chooses a particular function, for example, a polynomial, and utilizes the pixel values and relative positions to determine the function coefficients. Afterwards, the functions are evaluated at regular intervals to produce the voxel array (Figure 13). Rohling et al. [29] proposed the Radial Basis Function (RBF) that is an approximation with splines. The RBF should satisfy the smoothness requirement from an assumption that the input data is smooth at a scale of several B-scan pixels as well as the approximation requirement that comes from the existence of measurement errors. In order to efficiently increase the computed speed, the voxel array is divided into separated small, nonoverlapping rectangular segments where individual interpolating functions are calculated until all the voxel array is covered. An overlapping window that can be expanded sufficiently in all directions to encompass the pixels of the segment and the neighboring pixels is established to get smooth connections among the neighboring segments. All data inside the window is used to calculate the RBF for the enclosed segment and produce a continuous 3D result after all the segments have been traversed. Another popular algorithm called Rayleigh interpolation with a Bayesian framework estimates a function for the tissue by statistical methods where the Rayleigh distribution is to describe the US data. Sanches and Marques [46] further sped up the algorithm by running the first iterations on low resolution of the voxel volume.

Most recently, Huang et al. [47] have designed a fast interpolation method for 3D US with sparse scanning based on Bezier curve. They used a control window to cover 4 adjacent original frames and thus 4 pixel points at the same position on the 4 adjacent B-scans were set to be the control points to determine a 3rd-order Bezier curve. Then voxels located on the path in the reconstructed coordinate were interpolated. It can be described as the following formulas:

(4) Vt=P01−t3+3P1t1−t2+3P2t21−t+P3t3,t∈0,1,

where *V*(*t*) denotes the voxel value, *P*_0_, *P*_1_, *P*_2_, *P*_3_ are the 4 control points transformed from the corresponding 4 pixels, and *t* means the normalized distance from the voxel to *P*_0_.

**Figure 14:**
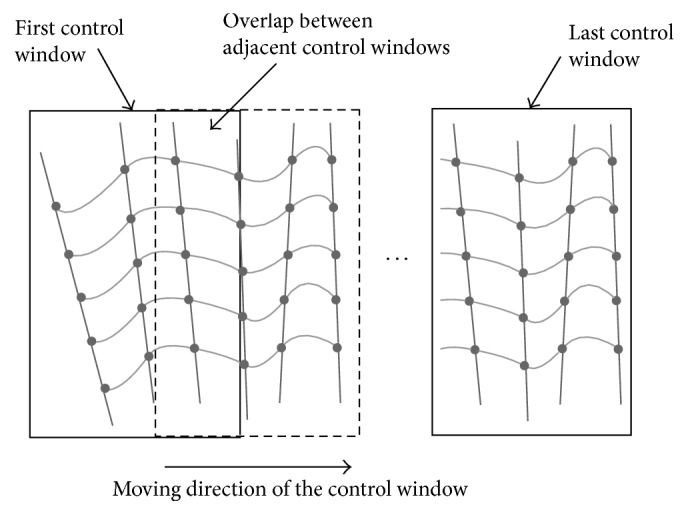
Bezier interpolation. Movement of the control window along with the sequences of B-scans for reconstruction of 3D volume.

After the voxels along the Bezier curves have been traversed, the control window moves to the next 4 adjacent B-scans along the scanning direction to repeat the voxel filling. In order to avoid gaps and make a continuous volume, the adjacent control windows are overlapped by 50% and voxels falling into the overlapped region get values by a distance weighted averaging strategy as follows:

(5) $$\begin{array}{c} V=\frac{d_{2}}{d_{1}+d_{2}}V_{pre}+\frac{d_{1}}{d_{1}+d_{2}}V_{cur}, \end{array}$$

where *V*_pre_, *V*_cur_ denote the voxel values that are calculated from the previous and current control window, respectively, and *d*_1_, *d*_2_ refer to the distance from the voxel to *P*_0_ of the current Bezier curve and *P*_3_ of the previous Bezier curve (Figure 14). The method can speed up the reconstruction mostly for a single 3rd-order Bezier curve using 4 control points is able to estimate more than 4 voxels whereas the estimation of a voxel value often requires a number of pixels in conventional techniques [47].

### 3.4. Computation Time

In terms of real-time 3D US system for practical clinic, like intraoperation, reconstruction and rendering speed are the most important aspects that should be taken into account. The reconstruction time of various algorithms is listed in Table 2. Since the raw B-scan data to be processed is different and the hardware differs in performance, the frame size, volume size, and hardware are included if possible to give a better comparison. From the table we can see that some of the algorithms reconstruct volume in real-time or near real-time (PTL, Bezier interpolation) while others need much long time (two adjacent frames' interpolation, RBF). It is obvious that the simple methods like VNN and PTL achieve a satisfying computation performance for they adopt plain process architecture. Those utilizing a neighborhood (i.e., 3D kernel) to achieve a more accurate reconstruction result increase the computation complexity, thus resulting in higher cost of computation time. For the kernel-based algorithms, computation cost can be reduced through minimizing the neighborhood size or selecting relative simple kernel and weighting (spherical kernel works faster than ellipsoid kernel; linear weighting performs better than the nonlinear weighting with spherical kernel). Although the RBF is declared to achieve encouraging reconstruction accuracy, it cannot be acceptable in most practical application for its intolerable computation time. Another function-based algorithm, that is, Bezier interpolation, however, performs the reconstruction closely to real-time as it takes advantage of Bezier curves to use 4 control points to interpolate more voxels in the path. It is claimed to achieve fast and accurate reconstructed result compared to the VNN and DW methods in processing sparse raw data for it can better track the changing trend of the control points [47]. It should be noted that although the algorithms in the table cannot reach a fully real-time effect (B-scan image acquisition rate is typically 25 or 30 frames/s), the algorithms can be accelerated to reach real-time.

With the increasing computation power of hardware or the parallel computing technique, successful stories have been reported using Graphics Processing Unit (GPU) to make the reconstruction completed in real-time [48]. Dai et al. [49] accelerated the incremental reconstruction up to 90 frames/s with a common GPU. Chen and Huang [50] implemented two real-time visualized reconstruction methods based on SDW and Bezier interpolations with the help of a common GPU, which speed up the frame rate from 1.33 frames/s and 20 frames/s to 32 frames/s and 119 frames/s. Considering that a powerful processor is always expensive, GPU that can be found in most PCs may be a suitable choice to speed up the reconstruction for the real-time visualization. Algorithms work on GPU must be parallelized. Thus, parallel performance is one of the important aspects in choosing a reconstruction algorithm, and luckily, most of the algorithms in Table 2 meet the requirement.

### 3.5. Reconstruction Quality

The reconstruction accuracy and display quality are also needed in 3D real-time US imaging for effective diagnosis. Various factors impact the final reconstruction quality, including the probe resolution, the rationality of reconstruction algorithm, probe calibration, and position sensor accuracy. Among these factors, we are likely to analyze how the algorithms impact the reconstructed result. A commonly used quantitative analysis method for reconstruction quality is the leave-one-out test, where some pixels from the raw data are removed before the remaining data are used to reconstruct the voxel array, and the reconstruction error is defined to the average absolute difference between the missing pixels and the corresponding reconstructed voxels [47]; that is,

(6) $$\begin{array}{c} E=\frac{1}{N}\overset{N}{\underset{i=1}{\sum }}\left|\;p_{i}-r_{i}\;\right|. \end{array}$$

Table 3 is extracted from [47] to show the averaged interpolation errors and the standard deviations for several reconstruction algorithms for reconstructing a fetus phantom and the data removing rate is 100%, that is, one frame. It may be a valuable comparison to detect the reconstruction quality of the three types of reconstruction algorithms.

VNN traverses the voxels in the grid and hence avoids the hole-filling stage, making it one of the fastest reconstruction algorithms. However, it seems to be the most inaccurate method compared to others in many published papers (Rohling et al. [29], Huang et al. [51]) for its inevitable drawback of introducing the most artifacts into the volume. The PNN outperforms the VNN as it allocates the pixel to the nearest voxel and a subsequent step is taken to fill the holes by combining pixel values in a neighborhood, making the volume continuous. Unfortunately, artifacts can be generated by this two-stage process, for example, the boundary between the highly detailed “nearest mapping” and the smoothed “hole-filling” voxels [29]. The VBMs and PBMs that apply a 3D kernel to the pixel (i.e., kernel-based algorithms) allow several pixels making contributions to the voxels in the neighborhood further and can improve the reconstruction accuracy [52]. Several parameters, for example, the shape of the kernel (spherical or ellipsoid), the size of the kernel, and the weighting type (linear and nonlinear inverse distance, Gaussian), influence the kernel-based methods' computation cost and reconstruction accuracy. It is shown that the ellipsoid Gaussian kernel outperforms the spherical kernel for it takes the asymmetric shape of point spread function of the US beam [29]. Nevertheless, it requires expensive computation compared to the spherical one for it introduces more complex neighborhood shape and weight function. It should be noted that the kernel-based methods can reduce the computation time through minimizing the neighborhood size but bring in more holes that need to be filled in the hole-filling stage. Moreover, if the size is set to be large, smaller gaps but excessive smoothing will occur. The most recent function-based algorithm called Bezier interpolation deserves our full attention for its best performance in processing sparse raw scan data. In intraoperation, the physician may scan fast to get an immediate feedback of the scanning region; thus, the acquired B-scan data is usually sparse [45, 53]. With the advantage of fast reconstruction with better reconstruction accuracy, the Bezier interpolation method will make a big count in clinical practices.

### 3.6. Real-Time versus High-Quality Reconstruction

Generally speaking, a high-quality reconstruction algorithm introducing more complex processing architecture that requires expensive computation may not be implemented in real-time with current common processors. In order to achieve the real-time goal, the simple methods designed to minimize the time and the memory required for reconstruction become a suitable choice. Nevertheless, with the increases in the computational power of PCs and the rapid development in parallel computing technique, it is full of possibility of completing the high-quality algorithms in real-time. By taking advantage of the large number of parallel executing cores in modern GPU [54], many researchers have used GPU as accelerators across a range of application domains [55], including the 3D US. Dai et al. [49] processed the PTL interpolation with compounding on the GPU in parallel and achieved a real-time reconstruction of up to 90 frames/s. Chen and Huang [50] performed the SDW interpolation on a common GPU and achieved a faster speed of 32 frames/s. Moreover, Chen and Huang [50] utilized the parallel computing on Bezier interpolation, which extremely accelerates the reconstruction speed at 119 frames/s. Hence, it is no doubt that GPU could be an ideal solution to settle the computational requirement in 3D US for a real-time goal.

## 4. Volume Rendering

### 4.1. Rendering Algorithms

The reconstruction speed and quality have a serious influence on the implementation of real-time visualization and the accuracy of practical diagnosis. However, the rendering technique also plays a significant and, at times, dominant role in transmitting the 3D information to the physicians timely. There exist three basic approaches for 3D visualization of US images: that is, slice projection, surface rendering, and volume rendering [56].

The slice projection allows users to view arbitrary slices from any angle of the scanned object. It can be real-time but still has the drawback that the physicians have to mentally reconstruct the 2D slices in 3D space [5]. Surface rendering based on visualization of tissue surfaces just simplifies the data set to rapidly describe the shapes of 3D objects such that the topography and 3D geometry are more easily comprehended [57]. In this approach, a segmentation or classification step is performed before rendering, losing some features of the data set, and making the method particularly sensitive to noise. Volume rendering displays the anatomy in a translucent manner. It allows physicians freely to choose the opacity values to selectively highlight particular features of the volume objects, which improves the diagnostic accuracy. Nevertheless, since every acquired data element influences every rendered view, this method requires expensive computation.

Both the slice projection and surface rendering only display a small part of the whole 3D information acquired at any one time. Due to the less computational requirement, many systems acquired the interactive rendering through slice projection in the past; for example, Prager et al. [25] rendered any slice of the scanned object using the Gouraud technique.

Volume rendering, however, preserves all the 3D information, making it the most common technique for 3D display [32]. Among volume rendering, the opacity-based ray-casting method is popularly used in 3D US display [58]. Thanks to the rapid development in computer technology, the method can be completed quickly, even in real-time. Therefore, we just give an outline of the opacity-based ray-casting volume rendering algorithms and put an emphasis on the rendering arrangement during volume reconstruction.

**Figure 15:**
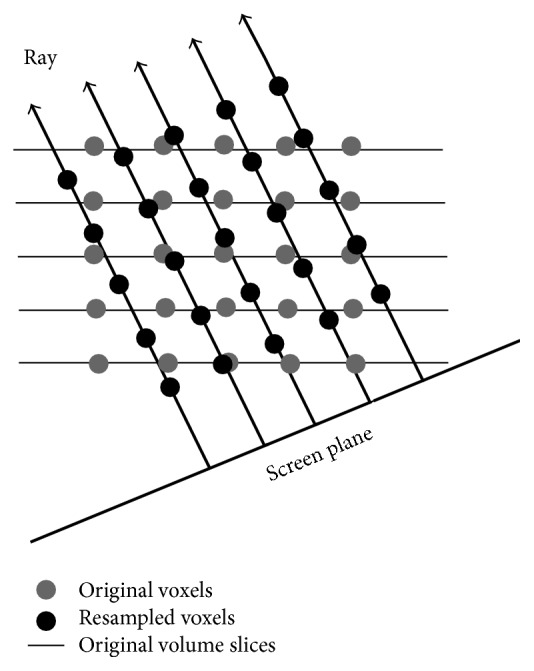
Volume rendering using direct ray casting. Voxels along each ray are resampled via a trilinear interpolation of eight neighboring original voxels.

One early approach for ray-casting volume rendering is based on intensity projection techniques [59]. It casts rays through the 3D image and every ray intersects the image with a series of voxels and then the voxel values are weighted or just picked the maximum value for each ray to show the anatomy in a translucent manner. A more realistic, opacity-based volume rendering technique based on optical models had been first proposed by Levoy [57] to delineate surfaces and convey depth information better (Figure 15). The rendering algorithm includes two main operations, that is, volume classification and shading. Volume classification assigns opacities to voxels in a volume dataset. Through a rational design of optimum opacity transfer functions, users can achieve high-quality rendering that makes structures of interest more prominent and the background structures less visible. Shading detects the surface orientation and assigns color to each voxel, depending on an illumination model and the surface orientation. After these two operations, a projection operation named as compositing casts rays from the pixels of the final present image plane into the volume to resample the voxels at equidistant intervals. The sampled voxels get opacities and colors through trilinear interpolation using the eight nearest voxels in the original volume grid and then the resampled opacities and colors are merged with each other and with the background by compositing to yield the final colors for the rays and since only one ray is cast per image pixel, for the corresponding pixels of the image plane.

**Figure 16:**
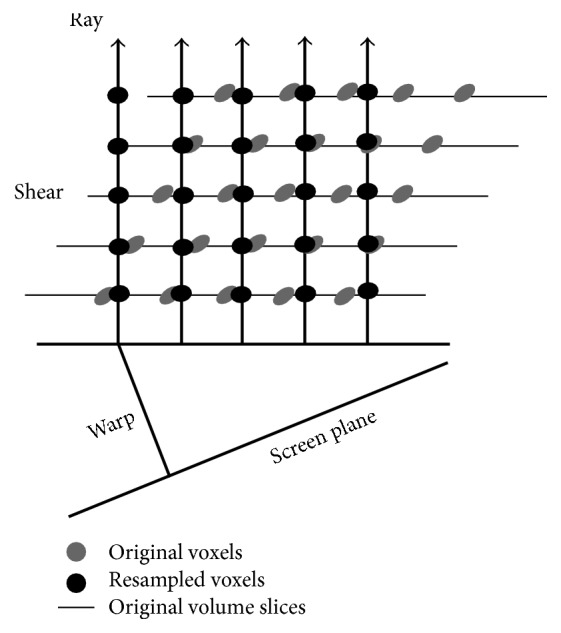
Fast volume rendering using shear-warp. Bilinear interpolation is used within each slice to resample each voxel along a ray from the four neighboring original voxels.

The conventional direct-ray-cast volume rendering has an inevitable drawback of incoherent data access, thus resulting in an inefficient computation since memory architectures suffer from long latencies in case of random accesses. Some efforts were made to overcome the high computational cost. One of the fastest classic algorithms that are designed to overcome the expensive computation in direct-ray-cast method is the shear-warp algorithm which breaks down ray casting into two stages [60], that is, the shear component and the warp component (Figure 16). It processes the 3D data slice by slice on the original volume grid to reduce the computationally expensive trilinear interpolation to bilinear interpolation and at the same time makes the data access coherent by confining the resampled voxels to one slice at a time. However, the confinement of voxel sampling locations to discrete slice locations results in aliasing in compositing and loss of sharp details occurs because of multiple stages of resampling.

**Figure 17:**
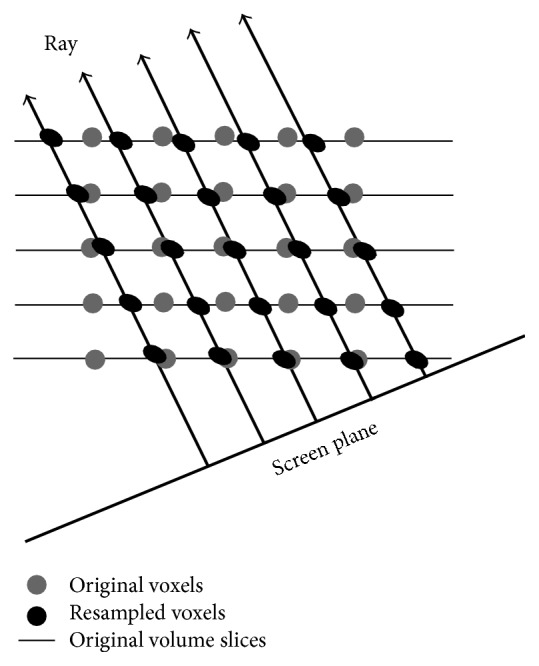
Fast volume rendering using shear-image-order. Bilinear interpolation is used within each slice to resample each voxel along a ray from the four neighboring original voxels.

Another limitation is the Venetian-blinds artifact on some viewing angles due to the volume shear that is difficult to remove completely [61]. Wu et al. [62] proposed the shear-image-order algorithm (Figure 17) mainly to overcome the problems associated with shear-warp. It eliminates the need for the final affine warp in the shear-warp algorithm through resampling each slice to make the interpolated voxels aligned with the pixels in the final image, preserving the sharp details better. Also, the shear-image-order algorithm makes each slice undergo a 2D shear to correct for the distortion resulting from restricting the sampling locations to original slice locations, which remains the shear-warp's data-access efficiency [61].

### 4.2. Rendering Arrangement

There are mainly two arrangements for rendering during data acquisition and insertion: one is to render the volume as each newly acquired image arrived and has been inserted into the voxel grid (i.e., slice-by-slice incremental volume rendering); the other is to wait for a fixed number of frames to be mapped onto the volume before the rendering.

#### 4.2.1. Multiple Scans per Rendering (MSPR)

Due to the heavy computation in rendering and limited computational capacities of common processors, it is reasonable to render the partial volume after several scans have been inserted into the grid array to obtain a near real-time visualized feedback. Several researchers have attempted this arrangement to achieve a near real-time result. Welch et al. [23] developed a system that updates the scanned volume with the capacity to simultaneously view cross-sections through the volume and a volume-rendered perspective view. The system waits for a fixed number of frames to form a data scan block which is then mapped onto the volume where the voxel value comes from the average of pixels at that position. The gaps are filled using the distance weighted average of the two nearest scanned voxel values in the scan direction. Then a rendering engine provided by CBYON, Inc., is applied to render the partial volume. The render rate reached roughly 15 frames/s as well as the reconstruction rate and thus, the system achieved a near real-time result. In addition to the qualitative feedback, that is, views of the partial volume, Dai et al. [26, 49] designed a system that provides real-time quantitative feedback on reconstruction, allowing the physicians to get hold of the process rate of reconstruction and determine when to terminate the scanning. In the system, pixels of every newly acquired B-scan are assigned into the predefined volume with the PNN method presented by Rohling et al. [29], and after insertion of the latest captured image, the reconstruction ratio (RR) and increased ratio (IR) are calculated. The RR is updated immediately to users to provide the quantitative feedback while IR is used to drive the volume rendering as the IR exceeds a predefined threshold. The researchers used a module provided by Medical Imaging Toolkit (MITK) for the rendering. The reconstruction and visualization are all performed on a personal computer. They set the IR threshold to 5% and achieved a visualization rate of 12.5 frames/s when the size of the reconstructed volume was 200 × 200 × 200 and the B-scan size was 552 × 274.

To implement the real-time visualization during the data acquisition and improve reconstruction accuracy, Chen and Huang [50] proposed a real-time freehand 3D US imaging system based on Bezier interpolation. Their method computes the incremental volume reconstruction, hole-filling, and volume rendering on a common GPU. As for the volume reconstruction, every five newly acquired B-scans are calculated by 4th-order Bezier interpolation kernel in their corresponding allocated device memory and interpolated into voxels. A C-function which executes the Bezier interpolation kernel for *M* times in parallel by different CUDA threads is called. To speed up the reconstruction, the block size was set to 32 × 32, while each thread processed 4 × 4 pixels of the image sequentially to reduce the insertion error. After the incremental reconstruction, some empty holes may exist. The hole-filling could be performed on the GPU, with the block grid set the same as the B-scan image number. In the volume rendering stage, a ray-casting volume rendering is used to render the 3D volume. Since the computation for composition is independent, each thread can deal with a subimage rendering in parallel. With the appropriate arrangement of reconstruction and rendering, utilizing the parallel computing of GPU can extremely accelerate the speed of visualization to 191 frames/s when the B-scan size was 302 × 268 and the volume size was 90 × 81 × 192.

#### 4.2.2. One Scan per Rendering (OSPR)

The OSPR means the arrangement of rendering the partial volume immediately after a newly captured B-scan has been inserted into the volume grid. Its feasibility has been demonstrated.

Ohbuchi et al. [34] developed a system to perform the rendering immediately after every newly acquired B-scan was reconstructed into the volume. They selected a finite 3D Gaussian kernel for incremental reconstruction and the rendering algorithm is an improved image-order, ray-casting version based on Levoy's [57]. The rendering method takes advantage of the incremental nature of the input and only the voxels in the proximity of the new 2D slice are sampled. It keeps the result of each incremental ray-sampling in a 3D array. Also, they reduced the compositing cost by introducing the idea of tree-structured Hierarchical Ray-Cache (HRC). The HRC stores the ray samples in its leaves and partially composites the results in its nonleaf nodes which can be reused in the compositing procedure. Due to the limited computational capacity of the workstation, the incremental reconstruction and rendering algorithm yielded a disappointing speed of no more than 1 frame/s.

Gobbi and Peters [33] used the Visualization Toolkits (VTK) to perform the 3D rendering. There were totally 5 threads in a 2 CPU 933 MHz Pentium III workstation to perform the data acquisition, reconstruction, and visualization. One thread waited for the tracking information of the scanner while the second thread moved each acquired B-scan onto a stack along with a time stamp. Two threads parallelized the interpolation of the most recent acquired frame; one performed the splats for the top half of the video frame and the other for the bottom half of the frame, and the reconstruction rate was 20 frames/s for PTL via alpha blending and 12 frames/s for PTL via compounding when the frame size was 320 × 240 and the volume size was 256 × 193 × 256. The main application thread rendered the partially reconstructed volume. Due to the high computational load on the computer and the limited process or speed, however, the rendering refresh rate was just 5 Hz.

In order to complete a fully interactive 3D US imaging system, Dai et al. [49] took advantage of the large number of parallel executing cores in a modern GPU to accelerate the incremental volume reconstruction and rendering. In the reconstruction, each captured B-scan was inserted into the volume using pixel 3D kernel interpolation in which the kernel was a 2 × 2 × 2 cube. Numerous threads in the GPU executed the incremental reconstruction kernel. A C-function defined in accordance with the NVIDIA Compute Unified Device Architecture (CUDA) inserted the image in parallel. Additionally, to avoid parallel insertion errors, each thread just processed 4 × 4 pixels of the image in order. In the volume rendering, they used ray casting to render the volume. Instead of performing ray casting for the entire display image every time a newly acquired B-scan was inserted into the volume. It just casted rays for pixels of the subimage that comes from the projection of the subvolume whose voxels were just updated from the most recent interpolation. There were two main steps to implement the incremental volume rendering. First, the position and size of the subimage in the projection image were figured out on the host and then sent to the device memory, that is, the GPU memory. Second, on the device, ray-casting kernel was called to update the subimage where each pixel was processed by a thread. Thanks to the rational arrangement of reconstruction and rendering and the powerful parallel computational capacities of the GPU, the system could provide real-time visualization feedback (over 25 frames/s) during data harvesting.

The foresaid real-time freehand 3D US imaging system proposed by Chen and Huang [50] could also take advantage of SDW interpolation, in which the incremental volume reconstruction, hole-filling, and volume rendering are calculated on a common GPU, similar to the Bezier interpolation method. However, the incremental kernel was called when every newly acquired scan arrives to perform insertion in parallel on the GPU. The block size was 32 × 32 to obtain a high speed while each thread managed 4 × 4 pixels of the acquired images. To avoid the insertion error, each thread processed all the pixels in the preset spherical neighborhood. Thanks to the parallel computing of GPU, the 3D volume could be reconstructed and displayed at 32 frames/s when the acquired B-scan size was 302 × 268 and the volume size was 90 × 81 × 192.

#### 4.2.3. OSPR versus MSPR

Since volume rendering needs a heavy load of computation, it is hard to achieve a real-time rendering in a common PC. Thus, an interactive 3D US system is hard to accomplish. However, it is possible to gain a near real-time effect if we choose a rational rendering arrangement during data acquisition. The OSPR arrangement can achieve a more smooth interaction with a higher time cost in rendering [33, 34]. The alternative method, that is, MSPR, yielding a better trade-off between computational cost and interactivity, can provide a better feedback [23, 26]. Nowadays, with a rapid development in the computer technology, the powerful computational capacity and even the mature parallel computing technique can help in speeding up the reconstruction and rendering processing. As we can see, some systems have utilized the parallel technique to achieve an interactive result. For instance, Dai et al. [49] have made use of the large amounts of parallel executing cores of GPU to perform the incremental reconstruction and rendering in real-time. Therefore it would be better to choose the OSPR rather than the MSPR arrangement for a fully interactive effect since GPU is more and more common in standard PCs and many of the reconstruction and rendering algorithms can be easily parallelized.

## 5. Applications

With the improvements in acquisition techniques, reconstruction algorithms, rendering methods, and computer GPU acceleration approaches, nowadays real-time 3D US imaging has been inundated in everyday clinical use. The advantages of simultaneous visualization and flexible operations contribute the expansion in areas of clinical application. Hereon, several promising clinical applications of real-time 3D US imaging are discussed.

### 5.1. Obstetrics

Fetuses remain challenging and difficult to evaluate due to their random motions, rapid heart rates, and maternal respiratory. To minimize these artifacts, patients should hold their breath during the data acquisition of the fetus. Real-time 3D US imaging enables a quick view of expected results while scanning and permits setting up or adjusting the gain while acquiring images. As a result, clinicians can react immediately to dynamic changes in fetal position.

Using a convex transabdominal 3D mechanical probe (3.5 MHz) and surface rendering, a real-time 3D US imaging is available for fetuses surrounded by sufficient amniotic fluid in prenatal diagnosis. Abnormalities of fetal face, for example, micrognathia and cleft lip, can be detected in real-time 3D US imaging. In addition, real-time 3D US imaging can also be applied to assist the diagnosis in rib anomalies, fluid accumulation, and abnormal spine curvature [63]. Utilizing matrix array transducer allows multimodality, for example, live xPlane imaging and live 3D surface, to examine the fetal heart in real-time [64].

### 5.2. Cardiology

To avoid estimation errors in geometrical assumptions and illusory displacement of the true boundary caused by out-of-plane cardiac motion when using traditional 2D US images, real-time 3D echocardiography was proposed to entirely visualize the anatomy of the cardiac chambers.

With the integration of matrix transducers, real-time 3D echocardiography is increasingly used to quantitatively measure left ventricular volume and dynamic changes of chamber volume during the cardiac cycle. It provides functional information, for example, blood flow and ejection fractions, to diagnose ischemic and congenital heart disease [65]. In addition, using a full volume probe is able to reconstruct large-range pyramid-shaped 3D images in near real-time [66].

Transthoracic and transesophageal approaches are both feasible for real-time 3D echocardiography [67]. Tridimensional myocardial structures can be obtained to examine anatomic defects. By utilizing stress echocardiography method, coronary artery disease can also be detected [68].

### 5.3. Surgical Guidance

Conventional 2D US imaging has limitation in locating the precise position in an oblique plane. With the advent of real-time 3D US imaging technique, the full visualization of the entire tissue with multiple transverse scans has become available. Making use of matrix array transducers, the real-time 3D transrectal US can improve the accuracy of the prostate implant [69]. Other interventions, for example, cryoprobe, which assists in treating prostate cancer and prostatic hyperplasia, can also take advantage of precise guidance from real-time 3D US imaging with a mechanical 3D probe in rotational scanning [70].

With the guidance of real-time 3D US imaging, biopsy is able to definitively diagnose cancer and reduce the psychological trauma in surgery [71]. Real-time 3D US imaging acquired by a matrix array transducer or a mechanical 3D probe in rotational scanning and rendered by slice projection or volume rendering method can assist clinicians to manipulate the needle tip to targeted lesion within the breast or nerve [72].

With the merit of perceived safety, portability, and dynamic imaging, 3D real-time US is capable of minimizing the surgical invasion, which facilitates it to be a useful guidance for intraoperative resection of gliomas [72] and brain tumors [73]. It is also widely utilized for monitoring abdominal radiation therapy [74] as well as regional anesthesia of femoral nerve [75] and lumbar spine [76].

### 5.4. Musculoskeletal Tissues

Real-time 3D US imaging can easily demonstrate anatomical details of small joints which are undetectable using traditional 2D US imaging and dramatically reduce the examination time. These advantages make real-time 3D US imaging apparently more suitable for musculoskeletal examination. Due to the variety of size and location of musculoskeletal structures, the transducer for 3D musculoskeletal US imaging should be appropriately selected considering different frequency. Transducers with high frequency are able to obtain high-resolution images. However, their penetrance is weaker, making them more suitable for superficial and small-size structures [77].

With the ability to image articular and periarticular structures, real-time 3D US imaging is increasingly applied into diagnosis of rheumatology. The detection of rheumatology includes bone erosions in small joints, enthesitis, and partial tear of tendons, which require the demonstrated images with high quality [78].

Making use of a mechanical localizer and parallel computing reconstruction, the forearm including bones, muscles, and connective tissues can be clearly visualized in near real-time [79]. Utilizing the mechanical 3D probes with PBMs, the 3D anatomy images of lumbar spine can be obtained and visualized in real-time as guidance in spinal needle injections. The epidural space and the facet joints are of significant interest among anatomical features. Using matrix array transducer can increase the processing speed and improve the image quality at the same time [80].

### 5.5. Vascular Imaging

Accurate assessment of vascular characteristics, for example, vessel anatomy and blood flow distribution, requires imaging technique capable of producing 3D images in real-time [81]. Real-time 3D US has capacity of not only noninvasively providing the anatomic geometry for numerical simulation of hemodynamics, but also demonstrating the dynamic 3D behavior of vessels [82], enhancing its wide applications in diagnosis of angiosis.

Taking advantage of a mechanical 3D probe or a freehand convex probe (1–5 MHz) combining with a magnetic position tracker, the measurement of aortic diameter, plaque volume, and stenosis degree can be implemented for predicting aortic aneurysm [83]. The aortic wall strains, which are indicators of biomechanical changes caused by aortic aneurysm, can also be detected by real-time echocardiography [84].

The arterial wall motion and hemodynamics are of great significance in early diagnosis of carotid atherosclerosis. With a linear mechanical 3D probe, the wall shear stress which is considered being related to development of atherosclerosis can be evaluated accurately [82]. As for blood flow velocity distribution, Doppler imaging [85] or matrix array transducers at a high volume rate (4000 volumes/s) [86] are generally utilized in clinics.

In addition, real-time 3D intravascular US imaging making use of an electromagnetic tracking sensor or an optical positioner enables the precise alignment of endovascular aortic stent grafting [87] and detection of peripheral blood vessels for cannula insertion guidance [88].

### 5.6. Urology

Real-time 3D US has been demonstrated as a noninvasive alternative to conventional voiding cystourethrography (VCUG), which is an invasive investigation for diagnosis and treatment monitoring of vesicoureteral reflux [89, 90]. Using the marker-based tracking methods, real-time 3D US is capable of navigation in urological surgery [91] and removing obstructions in urinary flow [92].

Prostate brachytherapy is considered as an effective treatment for early prostate cancer [93]. To confirm the success of the execution of punctures, the needle should be placed on the correct positions critically and rapidly [94]. Under the guidance of the 3D real-time transrectal US, which is mounted with a micro-magnetic sensor or an optical sensor [95], the preoperative oncological data as well as surrounding vital anatomies can be better understood and the precision of placing needles or catheters into the prostate gland has been well increased [96]. The same technique can also be applied to implement prostate biopsy [97] and quantify the prostate swelling [98].

Besides, real-time 3D US-based virtual cystoscopy imaging can be utilized to detect the bladder cancer recurrence [99]. Transurethral US (TUUS) imaging method is generally used in evaluation of the pelvic floor, urethra, detrusor, and levator ani. It provides useful information in diagnosis of stress urinary incontinence and etiology of pelvic floor dysfunction.

## 6. Conclusions

With the inherent nature of low cost [64] and no radiation, the capacity of dynamically visualizing the anatomy and geometry in real-time and user-friendly interaction with the operators expands the application of real-time 3D US imaging in clinical examinations increasingly. The main approaches to accomplishing a real-time US imaging system are systematically discussed in this review. The technical details of implementation and comparison among various approaches provide a guidance to design an appropriate system for practical use and improve the real-time 3D US imaging potentially with a higher quality and lower time cost. The usefulness of the real-time 3D US has been demonstrated by a large variety of clinical applications, further indicating its role and significance in the fields of medical imaging and diagnosis.

## Figures and Tables

**Table 1:** Performance and fabrication parameters of several typical real-time 2D array transducers.

Methods	Mill cross (17 × 17)	Mill cross (20 × 20)	T4 scanner (circular, *d*: 25 mm)	Receive multiplexing (256 × 256)	4Z1c matrix phased array	Imasonic SaS Voray (256 elements)
Transmitters	32	32	256	169	—	—
Receivers	32	32	256	1024	—	—
Frame rate	8 frames/s	8 frames/s	22 volumes/s	60 volumes/s	24 volumes/s	50 volumes/s
Scan view	65°	65°	64°	65°	—	90°
Frequency	1.7 MHz	1–2.3 MHz	2–3.5 MHz	5 MHz	2.8 MHz	4 MHz
Volume size	24 × 24	—	64 × 64 × 512	30 × 30 × 60	450 × 24 × 30	14 × 14 × 12
Resolution	4°	12 × 18	3 × 3 × 2 mm	—	2.5 mm	200 *μ*m
Authors	von Ramm et al. [100] (1990)	von Ramm et al. [101] (1991)	Stetten et al. [102] (1998)	Yen and Smith [6] (2004)	Frey and Chiao [103] (2008)	Deán-Ben et al. [104] (2015)

**Table 2:** Reconstruction rate of different reconstruction algorithms.

Algorithm reconstruction	Computation time	Input image size	Volume size	Hardware used	Reference
*VBMs*					
VNN	0.96 sec per image	302 × 268	200 × 90 × 330	3.0 GHz CPU	Sherebrin et al. [28] (1996)
Stradx	0.1 sec per any-plane slice	—	—	Regular PC in 2002	Prager et al. [25, 105] (1999, 2002)
DW	0.067 sec per image	—	—	500 MHz Pentium III workstation	Welch et al. [23] (2000)
Probe trajectory	0.8–2.6 sec per image	510 × 441	—	3.2 GHz Pentium	Coupé et al. [31] (2005)

*PBMs*					
PNN	0.033 sec per image	Cropped from 320 × 240	—	2 CPU 933 MHZ Pentium workstation	Gobbi and Peters [33] (2002)
PTL (kernel: 2 × 2 × 2)	0.05 sec per image (alpha blending) 0.08 sec per image (compounding)	Cropped from 320 × 240	—	2 CPU 933 MHZ Pentium workstation	Gobbi and Peters [33] (2002)
Spherical kernel (9 × 9 × 9) linear weighting	0.62 sec per image	640 × 480	205 × 175 × 273	2.4 GHz Pentium IV	Barry et al. [38] (1997)
SDW Spherical kernel (9 × 9 × 9) nonlinear weighting	0.75 sec per image	640 × 480	205 × 175 × 273	2.4 GHz Pentium IV	Huang et al. [39] (2005)
SDW Spherical kernel (9 × 9 × 9) nonlinear weighting	0.031 sec per image	302 × 268	90 × 81 × 192	3.4 GHz CPU 823 MHz GPU	Chen and Huang [50] (2016)
Ellipsoid kernel Gaussian weighting	1.5 sec per image	128 × 128	128 × 128 × 128	IBM RS6000 model 560 workstation	Ohbuchi et al. [34] (1992)
Ellipsoid kernel, off-plane: Gaussian in-plane: linear	2.6 sec per image	512 × 480	128 × 128 × 128	HP 9000/700 workstation	Ohbuchi et al. [34] (1992)
3D kernel	0.033 sec per image	480 × 413	256 × 256 × 256	1.86 GHz CPU 600 MHz GPU	Dai et al. [49] (2010)

*FBMs*					
Bezier interpolation	0.008 sec per image	302 × 268	90 × 81 × 192	3.4 GHz CPU 823 MHz GPU	Chen and Huang [50] (2016)

**Table 3:** The interpolation error of different reconstruction algorithms, and the scanned data is sparse.

VNN		DW		Bezier	
Mean	SD	Mean	SD	Mean	SD
13.32	1.69	11.29	1.26	10.58	1.38
